# High-Levels of Acquired Drug Resistance in Adult Patients Failing First-Line Antiretroviral Therapy in a Rural HIV Treatment Programme in KwaZulu-Natal, South Africa

**DOI:** 10.1371/journal.pone.0072152

**Published:** 2013-08-21

**Authors:** Justen Manasa, Richard J. Lessells, Andrew Skingsley, Kevindra K. Naidu, Marie-Louise Newell, Nuala McGrath, Tulio de Oliveira, the Southern African Treatment and Resistance Network (SATuRN)

**Affiliations:** 1 Africa Centre for Health and Population Studies, University of KwaZulu-Natal, Somkhele, South Africa; 2 Department of Clinical Research, London School of Hygiene and Tropical Medicine, London, United Kingdom; 3 MRC Centre of Epidemiology for Child Health, University College London Institute of Child Health, London, United Kingdom; 4 Academic Unit of Primary Care and Population Sciences, and Division of Social Statistics and Demography, University of Southampton, Southampton, United Kingdom; 5 Research Department of Infection, Division of Infection and Immunity, University College London, London, United Kingdom; University of California, San Francisco, United States of America

## Abstract

**Objective:**

To determine the frequency and patterns of acquired antiretroviral drug resistance in a rural primary health care programme in South Africa.

**Design:**

Cross-sectional study nested within HIV treatment programme.

**Methods:**

Adult (≥18 years) HIV-infected individuals initially treated with a first-line stavudine- or zidovudine-based antiretroviral therapy (ART) regimen and with evidence of virological failure (one viral load >1000 copies/ml) were enrolled from 17 rural primary health care clinics. Genotypic resistance testing was performed using the in-house SATuRN/Life Technologies system. Sequences were analysed and genotypic susceptibility scores (GSS) for standard second-line regimens were calculated using the Stanford HIVDB 6.0.5 algorithms.

**Results:**

A total of 222 adults were successfully genotyped for HIV drug resistance between December 2010 and March 2012. The most common regimens at time of genotype were stavudine, lamivudine and efavirenz (51%); and stavudine, lamivudine and nevirapine (24%). Median duration of ART was 42 months (interquartile range (IQR) 32–53) and median duration of antiretroviral failure was 27 months (IQR 17–40). One hundred and ninety one (86%) had at least one drug resistance mutation. For 34 individuals (15%), the GSS for the standard second-line regimen was <2, suggesting a significantly compromised regimen. In univariate analysis, individuals with a prior nucleoside reverse-transcriptase inhibitor (NRTI) substitution were more likely to have a GSS <2 than those on the same NRTIs throughout (odds ratio (OR) 5.70, 95% confidence interval (CI) 2.60–12.49).

**Conclusions:**

There are high levels of drug resistance in adults with failure of first-line antiretroviral therapy in this rural primary health care programme. Standard second-line regimens could potentially have had reduced efficacy in about one in seven adults involved.

## Introduction

South Africa has the largest HIV burden in the world, with an estimated 5.6 million people living with HIV [1]. The past eight years have seen massive scale-up of antiretroviral therapy (ART) in the country, which has substantially reduced population-level mortality and increased life expectancy [2], [3]. However, the number of people newly infected with HIV each year continues to exceed the number accessing ART [1]. In this context, antiretroviral drug resistance is a potential threat to the control of HIV [4].

South Africa follows the public health approach to ART delivery with standardised drug regimens and simplified decision-making, with the inclusion of routine viral load monitoring for the detection of treatment failure [5]. Viral load monitoring should enable early identification of treatment failure and, where appropriate, switch to second-line regimens. This has been shown to improve survival and health [6], [7]. Delay in switching to second-line therapy and prolonged viraemia compromise the response to standardised second-line regimens [8]–[11].

The majority of studies from South Africa focused on acquired drug resistance (resistance to one or more drugs in an individual who has been treated with antiretroviral therapy) have been conducted in urban, hospital-based treatment programmes [12]–[23]. There is a critical need for data from programmes in rural South Africa (distinction between urban and rural as defined by the South African Population Census 2011) [24], as there are many challenges unique to rural communities, and rural health systems remain critically under-resourced [25]. Here, we present data from a large, decentralised, primary health care HIV treatment programme in rural KwaZulu-Natal.

## Methods

### Ethics Statement

The study was approved by the Biomedical Research Ethics Committee of the University of KwaZulu-Natal (ref. BF052/10) and the Health Research Committee of the KwaZulu-Natal Department of Health (ref. HRKM 176/10). Written informed consent was obtained from all the study participants.

### Setting

The Hlabisa HIV Treatment and Care Programme is a decentralised, primary health care (PHC) programme in the predominantly rural Hlabisa health sub-district in northern KwaZulu-Natal. Details of the programme have been reported previously [26], [27]. HIV treatment and care is delivered at 17 primary health care clinics and one district hospital and is largely provided by nurses and counsellors, with weekly or fortnightly visits by a medical officer. All treatment and care is provided free of charge. The programme was supported from 2004 to 2012 by the US Agency for International Development (USAID) through the President’s Emergency Plan for AIDS Relief (PEPFAR).

The programme adheres to the national antiretroviral treatment guidelines [28], [29]. From the inception of the programme in 2004 until early 2010, first-line ART regimens were stavudine (d4T), lamivudine (3TC), and either efavirenz (EFV) or nevirapine (NVP). Viral load was measured every six months (repeated after three months if >5000 copies/ml), and a switch to second-line therapy was recommended if two consecutive viral loads (VL) were >5000 copies/ml despite good (>80%) adherence. Substitution of zidovudine (AZT) for d4T was allowed in the event of treatment-limiting toxicity [28]. In 2010, the frequency of VL monitoring was modified: measurement at month 6, month 12, then every 12 months if VL <400 copies/ml. Viral load >1000 copies/ml prompted repeat measurement after three months (including intensive adherence counselling) and the threshold for switch to second-line regimen was changed to two consecutive viral loads >1000 copies/ml. Tenofovir (TDF) also replaced d4T in first-line regimens and was available for substitution for individuals experiencing toxicity with d4T or AZT [29].

### Study Design

This was a cross-sectional study enrolling HIV-infected adults with virological failure on first-line antiretroviral therapy. Inclusion criteria were: adult (≥18 years); initiated on first-line d4T- or AZT-based regimen; received treatment for at least 12 months; and evidence of virological failure (defined for the purposes of this study as one viral load >1000 copies/ml). Exclusion criteria were: prior use of nucleoside reverse transcriptase inhibitor (NRTI) monotherapy or dual therapy (not including regimens for the prevention of mother-to-child transmission (pMTCT)), prior protease inhibitor (PI) use, and initiated on first-line TDF-based regimen. The decision to include only those who commenced treatment with d4T- or AZT-based regimens, and exclude those who initiated TDF-based regimens, was primarily to allow formal comparison with other studies in the region.

Individuals were recruited at all 17 PHC clinics between December 2010 and March 2012. There were two possible routes for enrolment: i) Routine clinic - adults with virological failure (latest VL >1000 copies/ml) were identified by clinic staff during routine visits and referred to the physician for review; ii) virological failure camp – eligible adults were proactively identified through the programme’s operational database, were contacted by programme staff, and were booked for physician review on a specific day at their regular clinic. In both models, the physician performed a clinical evaluation and obtained written informed consent for the study. A 5 ml EDTA whole blood sample for HIV drug resistance genotyping was collected during the clinical evaluation. Basic clinical and demographic data were collected on a standardised clinical form in parallel to the records in the Africa Centre’s ART Evaluation and Monitoring System (ARTemis), an operational database holding treatment and laboratory monitoring information. The clinical information was entered in anonymised form into a relational database, the SATuRN REGA database [30].

### Genotypic Resistance Testing

Specimens were transported daily from the clinics to the Africa Centre and the same day to the Africa Centre laboratory in Durban (200 km from site). At the laboratory, plasma was aliquoted and stored at −80°C until sequenced. Samples were sequenced within a week of collection. HIV RNA was extracted using the QIAMP RNA extraction kit (Invitrogen) modified to extract RNA from 200 µl of plasma instead of 140 µl, to concentrate the viral RNA for better amplification rates.

A previously described in-house HIV-1 drug resistance genotyping method was used to genotype the samples [31]–[33]. Briefly, the extracted RNA was reverse transcribed using the Superscript III 1^st^ strand synthesis kit (Life Technologies, Foster City, CA) followed by nested PCR using Platinum Taq polymerase (Life Technologies, Foster City, CA). Successful PCR amplification was assessed using 1% agarose gel (Bioline, Taunton, Massachusetts) electrophoresis run at a 100 V for 40 minutes. The PCR products were cleaned up using the PureLink QUICK PCR Purification Kit (Life Technologies, Foster City, CA) and sequenced using the Big Dye Terminator kit ver3.1 (Life Technologies, Foster, City) and a set of four bidirectional primers. Capillary sequencing electrophoresis was done on 3130Xl Genetic Analyzer (Life Technologies, Foster, CA).

The sequences covering all of the 99 protease codons and the first 300 codons of the reverse transcriptase region were assembled using Geneious Pro genetic analyzer [34]. The quality of the sequences was assessed using the HIV-1 Quality Analysis Tool [35] and the Calibrated Population Resistance (CPR) tool [36]. HIV-1 subtyping was performed using the REGA HIV-1 Subtyping Tool v 2.0 [37]. Phylogenetic analysis was done to aid with quality assurance of the sequencing. The sequences were aligned to a reference dataset of HIV-1 subtype C that included other sequences previously sampled in KwaZulu-Natal and sequences from other geographic regions (*n* >1000) available from public HIV sequence databases. Phylogenies were constructed using neighbour-joining (NJ), maximum likelihood (ML) and Bayesian methods. Reliability of the trees was assessed by bootstrap methods (1000 replicates) for NJ and ML. Posterior probabilities were calculated from a sample of 10 000 trees sampled over 1×10^7^ generations to determine the reliability of Bayesian trees. Trees were visualized using FigTree (http://tree.bio.ed.ac.uk/software/figtree). All the sequences generated for this manuscript are available from Genbank with accession numbers KC951632–KC951853.

The method was validated in using a panel of proficiency testing samples obtained from the French National Agencies for Research on AIDS and Viral Hepatitis (ANRS). The panel was analysed using the in-house method and the United States food and drugs Agency (FDA) approved Viroseq genotyping method in parallel. The laboratory participates in the Quality Control for Molecular Diagnostics (QCMD) proficiency testing programme, and receives one EQA panel per year. This validated method is an open access and discounted method, with reagent costs of about US$50, made available by a partnership between SATuRN and Life Technologies to laboratories in Africa. All resistance results were provided within 15 days to the physician and were used for clinical management.

### Data Analysis

The sequence data were linked to the clinical and demographic data in the SATuRN REGA database. The interpretation of drug resistance data from the nucleotide sequences was done using the Stanford HIVSeq algorithm version 6.0.5 [38]. Genotypic susceptibility scores (GSS) were calculated, also using the Stanford HIVSeq algorithm version 6.0.5, for each antiretroviral agent and then a total score was calculated for the standard second-line regimens. This was done to assess the impact of observed drug resistance mutations on the predicted effectiveness of standard second-line regimens. Total GSS for the standard second-line regimen was calculated depending on the patient’s treatment history: for participants on d4T or AZT at the time of genotyping, GSS was calculated for a regimen of TDF, 3TC and lopinavir/ritonavir (LPVr); whilst for those on TDF at the time of genotyping, GSS was calculated for a regimen of AZT, 3TC and LPVr. These standard second-line regimens were consistent with the recommendations in the current national ART guidelines [29]. For the purposes of this analysis, compromised second-line regimen was defined as GSS<2.

Age was defined as at the date of enrolment. Baseline CD4+ cell count was defined as the CD4+ cell count closest to but prior to the date of ART initiation. CD4+ cell count and viral load at time of genotype were the measurements closest to but prior to the date of genotype. Immunological failure was defined according to WHO guidelines: fall of CD4+ cell count to baseline or below; 50% fall from on-treatment peak value; or persistent CD4+ cell count below 100 cells/µl [39]. Duration of antiretroviral failure was estimated from the date of the first viral load >1000 copies/ml to date of genotype, unless there was a viral load <50 copies/ml in-between in which case the time was estimated from the next viral load >1000 copies/ml. If there was no viral load ≤1000 copies/ml then time was calculated from date of ART initiation.

All statistical analyses were performed using STATA version 11.2 (StataCorp, College Station, Texas). Descriptive statistics were used to summarise the baseline demographic and clinical characteristics. Frequency distributions of specific mutations were calculated. Logistic regression was used to explore factors associated with a compromised second-line regimen (GSS<2). The variables used for regression analysis included demographic characteristics (age and sex), laboratory results (CD4+ cell count and viral load), and treatment information (regimen, ART substitutions, duration of treatment).

## Results

**Figure 1:**
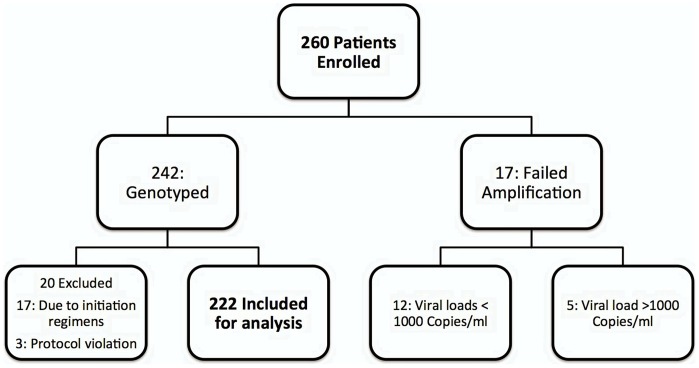
Flow chart showing patients excluded from analysis. Of the 20 excluded patients, 17 were initiated on TDF-based first-line therapy, dual NRTI therapy or were already receiving a second-line regimen at time of genotype. The three protocol violations were: two patients on treatment for less than a year, and one with viral load <1000 copies/ml at the time of genotyping.

A total of 260 individuals with virological failure on first-line ART were enrolled between December 2010 and March 2012. Samples from 242 (93%) of the patients were successfully genotyped and 222 (85%) were included in the analysis (Figure 1). All the 222 patients were infected by HIV-1 subtype C viruses. Of the 222 patients, 160 (72%) were women, and the median age was 37 years (IQR 32–44). One hundred and twenty-four (56%) were enrolled through the virological failure camp system, and 98 (44%) through the routine clinic system. The two enrolment groups did not differ substantially in sex, age, baseline CD4+ cell count, time on ART, time on failing regimen, or history of drug switch. Details of the demographic and clinical characteristics are summarised in Table 1.

**Table 1:** Demographic and clinical characteristics.

Characteristic	
**Sex, ** ***n*** ** (%)**	
Female	160 (72%)
**Age, years**	
Median (IQR)	37 (32–44)
18–24	8 (4%)
25–34	69 (31%)
35–44	94 (42%)
≥45	51 (23%)
**Baseline CD4+ cell count, cells/µl** a	
Median (IQR)	108 (50–169)
<50	48 (25%)
50–100	41 (21%)
101–200	88 (45%)
>200	17 (9%)
**CD4+ cell count at time of genotype, cells/µl**	
Median (IQR)	221 (124–322)
<50	16 (7%)
50–100	29 (13%)
101–200	54 (24%)
>200	123 (55%)
**Immunological failure at time of genotype, ** ***n*** ** (%)** b	75 (34%)
**Viral load at time of genotype, log_10_ copies/ml**	
Median (IQR)	4.25 (3.68–4.83)
**Time between last viral load and genotype, months**	
Median (IQR)	3.3 (1.5–6.0)
**Ever achieved virological suppression, ** ***n*** ** (%)**	
Viral load <1000 copies/ml	134 (60%)
Viral load <50 copies/ml	89 (40%)
**Duration of antiretroviral therapy, months**	
Median (IQR)	42 (32–53)
**Duration of antiretroviral failure, months** c	
Median (IQR)	27 (17–40)
**Initial antiretroviral regimen, ** ***n*** ** (%)**	
d4T/3TC/EFV	156 (70%)
d4T/3TC/NVP	64 (29%)
AZT/3TC/EFV	2 (1%)
**Antiretroviral regimen at time of genotype, ** ***n*** ** (%)**	
d4T/3TC/EFV	114 (51%)
d4T/3TC/NVP	53 (24%)
AZT/3TC/EFV	19 (8%)
AZT/3TC/NVP	4 (2%)
TDF/3TC/EFV	24 (11%)
TDF/3TC/NVP	8 (4%)
**Previous antiretroviral treatment substitution, ** ***n*** ** (%)**	
NRTI substitution	52 (23%)
NNRTI substitution	34 (15%)

IQR, interquartile range; d4T, stavudine; 3TC, lamivudine; EFV, efavirenz; NVP, nevirapine; AZT, zidovudine; TDF, tenofovir; NRTI, nucleoside/nucleotide reverse-transcriptase inhibitor; NNRTI, non-nucleoside reverse-transcriptase inhibitor.

a Baseline CD4+ cell count was measurement closest to but prior to ART initiation; 28 missing baseline CD4+ cell count.

b Immunological failure was defined according to WHO guidelines: fall of CD4+ cell count to baseline or below; 50% fall from on-treatment peak value; or persistent CD4+ cell count <100 cells/µl.

c Duration of antiretroviral failure was estimated from the date of the first viral load >1000 copies/ml to date of genotype, unless there was a viral load <50 copies/ml in-between, in which case the time was estimated from the next viral load >1,000 copies/ml. If there was no viral load ≤1,000 copies/ml then time was calculated from date of ART initiation.

Median duration of ART was 42 months (IQR 32–53) and median duration of antiretroviral failure was 27 months (IQR 17–40). One hundred and thirty four (60%) had achieved virological suppression (VL<1000 copies/ml) on at least one occasion prior to genotyping, but only 89 (40%) had achieved VL<50 copies/ml. Two hundred (90%) of the patients had two or more viral loads >1000 copies/ml before genotyping and the median number of viral loads >1000 copies/ml before genotyping was 3 (IQR 2–5). During therapy, 81 (36%) had one or more drug substitution, the majority of which were NRTI substitutions. At the time of genotype, 75 (34%) had evidence of immunological failure, according to WHO definitions.

**Figure 2:**
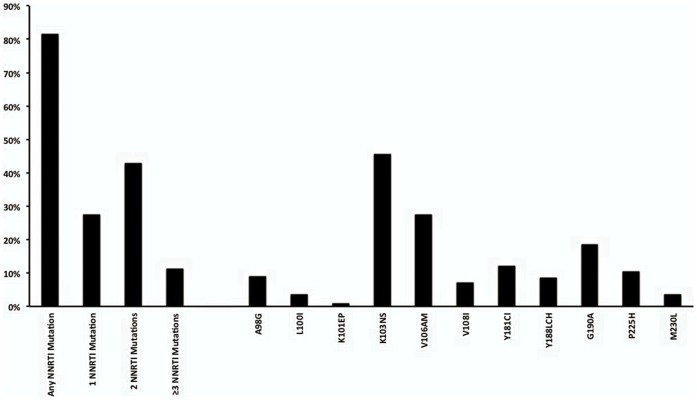
NNRTI mutations.

**Figure 3:**
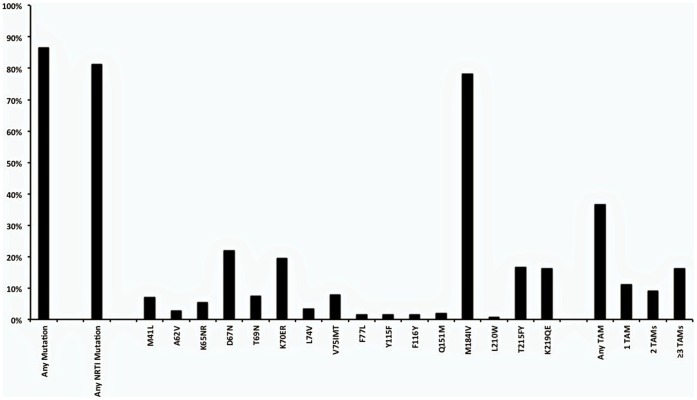
NRTI mutations, including summary of proportion with thymidine analogue mutations (TAMs).

One hundred and ninety one (86%) individuals had at least one drug resistance mutation at the time of genotyping: 181 (82%) had NNRTI resistance mutations and 179 (81%) had NRTI resistance mutations (Figures 2 and 3). M184V mutation was the most common mutation, detected in 173 (78%) patients. The K103N/S mutation was the most common NNRTI mutation, detected in 101 (45%) patients. Thymidine analogue mutations (TAMs) were detected in 88 (40%) of the patients and 39 (18%) had three or more TAMs (Figure 3). The K65R mutation was identified in 13 (6%) patients, of which eight were on TDF at the time of genotyping. The Q151M complex (Q151M, V75I, F77L and F116Y) was detected in 3 (1%) patients. One of these patients had the Q151M complex and K65R, resulting in high-level resistance to all NRTIs.

Thirty-four individuals (15%) had a calculated GSS <2 for the standard, guideline-recommended second-line regimen, suggesting a potentially compromised regimen. Five of these had a GSS of 1, suggesting that only the protease inhibitor would have full activity in the standard second-line regimen. The majority had a GSS of 2 (n = 143, 64%). Logistic regression was used to identify factors associated with compromised second-line regimen, defined as GSS <2 (Table 2). In univariate analysis, there was strong evidence that individuals with a prior NRTI substitution were more likely to have a GSS <2 than those maintained on the same NRTIs throughout (OR 5.70, 95% CI 2.60–12.49). There was weak evidence that males were more likely to have GSS <2 than females (OR 1.84, 95%CI 0.85–3.99). There was insufficient power to explore multivariable models given the limited number with GSS <2.

**Table 2:** Univariate analysis of factors associated with compromised standard second-line regimen (genotypic susceptibility score <2).

Characteristic	*N*	% GSS <2	Univariate OR (95% CI)	*p*-Value
**Sex**				
Female	156	13%	1.00	0.13
Male	61	21%	1.84 (0.85–3.99)	
**Age, years**				
18–24	8	25%	1.53 (0.27–8.51)	0.11
25–34	67	18%	1.00	
35–44	92	9%	0.44 (0.17–1.14)	
≥45	50	22%	1.29 (0.52–3.23)	
**Baseline CD4+ cell count, cells/µl** a				
<50	47	30%	1.00	0.009
50–100	41	15%	0.40 (0.14–1.18)	
101–200	86	8%	0.21 (0.08–0.56)	
>200	17	6%	0.15 (0.05–1.22)	
**Enrolment group**				
Routine clinic	95 (14%)		1.00	
Virological failure camp	122 (16%)		1.24 (0–58–2.64)	0.58
**Ever achieved virological suppression <1000 copies/ml**				
No	85	18%	1.00	0.42
Yes	132	14%	0.74 (0.35–1.56)	
**Ever achieved virological suppression <50 copies/ml**				
No	129	18%	1.00	0.19
Yes	88	11%	0.59 (0.27–1.31)	
**Duration of antiretroviral therapy, months**				
<24	20	20%	1.00	0.76
24–48	118	14%	0.63 (0.19–2.12)	
>48	79	16%	0.79 (0.23–2.74)	
**Duration of antiretroviral failure, months**				
<6	14	7%	1.00	0.30
6–12	21	5%	0.65 (0.04–11.33)	
13–24	63	17%	2.75 (0.33–23.27)	
>24	119	17%	2.62 (0.32–21.23)	
**Initial antiretroviral regimen**				
d4T/3TC/EFV	155	17%	1.00	0.30
d4T/3TC/NVP	62	11%	0.63 (0.26–1.54)	
**Previous NRTI substitution**				
No	167	9%	1.00	<0.001
Yes	50	36%	5.70 (2.60–12.49)	
**Previous NNRTI substitution**				
No	185	17%	1.00	0.07
Yes	32	3%	0.15 (0.02–1.17)	

GSS, genotypic susceptibility score; OR, odds ratio; d4T, stavudine; 3TC, lamivudine; EFV, efavirenz; NVP, nevirapine; NRTI, nucleoside/nucleotide reverse-transcriptase inhibitor; NNRTI, non-nucleoside reverse-transcriptase inhibitor.

a 28 missing baseline CD4+ cell count.

## Discussion

This study assessed the levels of acquired drug resistance in adults with virological failure on first-line ART in a rural primary health care treatment programme. This is one of the largest adult drug resistance studies to date in South Africa and the first to focus on a single rural treatment programme. In common with other studies from South Africa, almost all individuals with virological failure on first-line ART had evidence of antiretroviral drug resistance. In the context of public health antiretroviral strategies based on standardised first- and second-line regimens, it is important to explore how drug resistance impacts not only on individual antiretroviral drugs but also on drug regimens. Of concern was the fact that one in seven of these adults had complex resistance patterns with the potential to limit the efficacy of the standard second-line ART regimen. Of equal concern were the long periods of time on failing regimens despite the use of viral load monitoring, which suggest critical deficiencies in programme quality.

It has been well documented that prolonged failure on first-line regimens leads to the accumulation of drug resistance and to poorer outcomes on second-line therapy [8]–[10]. In this study we found 15% had three or more TAMs and 1% had the Q151M complex, patterns known to develop during long periods on a failing regimen [40], [41]. The presence of three or more TAMs (inclusive of M41L or L210W) significantly reduces the activity of TDF and thus a standard second-line regimen of TDF/3TC/LPVr might have suboptimal efficacy [42]. The proportions of TAMs reported here are broadly similar to those from hospital-based programmes in Johannesburg and Durban [17], [20], [22]. Conversely, they are higher than reported from the Western Cape, from Soweto and a workplace programme in Johannesburg [15], [16], [18], [21]; however, it should be noted that in two of those studies the threshold for definition of virological failure and for genotyping was lower at a single viral load >400 copies/ml (Table 3) [15], [21]. Although our definition was a single viral load >1000 copies/ml, almost all cases had two or more consecutive viral loads above this threshold prior to genotyping and so would have been eligible for a switch to second-line therapy according to national guidelines.

**Table 3:** Summary of acquired drug resistance studies in adults treated with first-line antiretroviral therapy in South Africa.

	Dates	Criteria	N	ART duration (months)	≥1 DRM (%)	NNRTI (%)	M184V (%)	TAM (%)	TAM ≥3 (%)	K65R (%)	Q151M (%)	Ref
Limpopo (rural clinic)	-	1×VL >1000	21	9.0	90.5	85.8	52.4	0.0	0.0	0.0	0.0	[13]
Durban (two urban hospitals)	Jun-05	1×VL >1000	115	10.8	83.5	78.3	64.3	32.2	13.0	2.6	0.9	[17]
Cape Town (eight urban clinics)	Jul-02	1×VL >1000	110	8.9	88.2	88.2	78.2	22.7	NR	9.1	0.0	[18]
Johannesburg (urban workplaceclinic)	Aug-02	1×VL >1000	68	–	66.2	61.8	36.8	5.9	NR	0.0	0.0	[16]
Johannesburg (urban hospitals)	–	2×VL >1000 or 2×VL >5000	226	–	83.0	77.9	72.1	31.0	12.0	3.5	2.2	[22]
Soweto (urban hospital)	2008	ART >12M & VL >400	94	–	80.8	80.8	61.7	16.0	NR	1.1	0.0	[15]
Johannesburg (urban hospital)	Sep-06	2×VL >5000	43	22.0	88.4	86.1	74.4	53.5	16.3	7.0	2.3	[20]
Western Cape (urban hospital& CHC)	Oct-07	1×VL >400	167	13.5	83.0	82.0	60.5	12.0	2.4	4.2	0.0	[21]
Johannesburg & Cape Town (urban clinical trial)	–	2×VL >1000	83	8.5	73.0	71.0	57.0	1.0	NR	3.0	0.0	[23]
Soweto (urban hospital)	2008	ART >12M & VL >400	38	45	81.6	81.6	65.8	21.0	10.5	2.6	0.0	[14]
Durban (urban hospital)	–	1×VL >5000	43	29	95.0	95.0	87.0	55.0	NR	NR	NR	[12]
Hlabisa (rural clinics)	**Dec-10**	**1**×**VL >1000**	222	42	86.0	83.0	78.0	40.0	18.0	6.0	1.0	–

ART, antiretroviral therapy; CHC, community health clinic; DRM, drug resistance mutation; TAM, thymidine analogue mutation; VL, viral load.

Missing values are where the relevant data were not presented in the manuscript.

The K65R mutation was present in 6% of cases, although over half of these were on TDF at the time of genotyping, due to previous substitution from d4T or AZT. There is evidence that K65R develops more frequently in subtype C viruses, primarily due to a difference in the template nucleotide sequence [43], [44]. K65R confers high-level resistance to TDF and its presence during failure of d4T-based treatment would therefore also compromise the activity of the standard second-line regimen.

Surprisingly, duration on ART and duration on failing regimen were not associated with more complex resistance patterns in this study population. This may partly be due to lack of statistical power but also potentially to the complex relationship between adherence and resistance [45]. The only adherence data available was the data in the clinical records collected using standard adherence assessment tools, as contained within the national ART guidelines [29]. However, the poor performance of self-reported adherence measurements has been previously reported from this programme [46]. About one in four individuals had a history of NRTI substitution, most commonly from stavudine to zidovudine. The observation that this was associated with GSS<2 may reflect the effect of poorer adherence due to debilitating long-term toxicities, such as peripheral neuropathy and lipodystrophy syndrome, with consequent accumulation of resistance in this subgroup. Furthermore, single drug substitutions could have occurred in the absence of viral suppression (or absence of recent viral load), thus compromising the modified regimen.

In this high prevalence community, there has been rapid scale-up of ART over the past eight years and high levels of ART coverage have been achieved in the community [27], [47], [48]. In this context and with on-going high incidence in the area [49], the high levels of acquired drug resistance give rise to the potential for transmitted drug resistance, although to date we have found no evidence of transmitted antiretroviral resistance in this community [31]. Prior to this study, despite more than 18 000 adults having started ART and relatively high rates of virological failure, remarkably few people (fewer than 50) had been switched to second-line regimens. This study was implemented not only to assess the levels and patterns of drug resistance but also to aid clinical management, and to focus attention and improve awareness of these issues within the programme. The comprehensive format for reporting resistance results with management recommendations to the clinic also provided a useful training tool for junior physicians, nurses and counsellors.

The national antiretroviral treatment guidelines recommend routine viral load monitoring to identify virological failure. However, if the results are not appropriately acted on and individuals are maintained on failing regimens, there may be accumulation of further resistance and progression to immunological and clinical failure. In this study, the period of time spent on failing first-line regimens was excessively long. Similar findings of prolonged viraemia have recently been reported from a multi-centre programme in Johannesburg [50]. Conversely, the IeDEA-Southern Africa collaboration reported a median delay of 4.6 months between identification of virological failure and switch to second-line therapy [51]. The five cohorts included in that analysis were mostly physician-led and well-resourced, hospital based programmes, which might not be representative of most South African programmes. There is clearly a need for more stringent adherence to the current monitoring guidelines and continued training of health care workers, but also a need to understand the programmatic factors that contribute to our findings. The priority both from the Department of Health and the funding agencies providing support in this area has understandably been initiation of ART for eligible individuals but as a result there has been much less effort directed at support and maintenance of people on lifelong ART [52]. The National Strategic Plan for 2012–2016 does highlight the need for ‘strengthening quality standards and adequate monitoring of drug resistance’ although it is notable that there is no explicit mention of monitoring virological outcomes in the monitoring and evaluation framework [53].

Health systems, particularly in rural areas, have been put under huge strain by the rapid scale-up of HIV testing, treatment and care. There is already evidence from South Africa that the quality of treatment programmes has declined as systems have become more stretched [54], [55]. One study in particular from Cape Town demonstrated an increasing risk of virological failure with each year of programme scale-up [55]. Adherence monitoring tools, predominantly reliant on patient self-reporting, have been shown to perform poorly [46]. There are also few adherence interventions of proven efficacy and so health care workers can find it difficult not only to identify but also to address adherence problems [56]. Antiretroviral therapy is often interwoven into complex lives and there are often multiple barriers to adherence, many of which are difficult, if not impossible, to overcome [57], [58].

Interpretation of these results is subject to a number of limitations. The study involved programme physicians recruiting cases as part of routine clinical care and constraints in the number of physicians meant that not all potentially eligible individuals could be recruited into the study. From programmatic data, as of 31 August 2012, approximately 10% (*n* = 930) of the adults active on first-line ART and with a viral load measurement beyond 12 months had a latest viral load >1000 copies/ml; the individuals included in this study represent around 30% of that total. We cannot therefore be certain that the enrolled individuals were representative of all adults with virological failure in the programme. There was a lack of reliable and appropriately measured indicators of individual-level adherence and also no information available from pharmacy records. The absence of baseline genotyping led to assumptions that all observed drug resistance were acquired during treatment, but this is likely to be a reasonable assumption given that we have found no evidence as yet of transmitted drug resistance in this community. The definition of compromised second-line regimen was based purely on the calculated GSS and we are prospectively following the cohort of individuals switched to second-line therapy to explore clinical outcomes on second-line therapy.

In summary, there are high levels of acquired drug resistance in adults with failure of first-line antiretroviral therapy in this rural programme. Whilst the levels of resistance are similar to those reported from other programmes in South Africa, the long periods of antiretroviral failure reported here give cause for concern. The transition from an emergency response to the HIV epidemic to a sustainable, long-term solution presents many challenges [59], [60]. Management of increasingly complex drug-resistant cases through the public health system is difficult and so programmatic strategies for prevention and management of drug resistance are critical. Continuous education, training and support for health care workers, and monitoring of performance in following guidelines are key components of any such strategy. Genotypic resistance testing could be important in future strategies to prevent and manage drug resistance.
